# Relation between the food environment and oral health—systematic review

**DOI:** 10.1093/eurpub/ckac086

**Published:** 2022-07-18

**Authors:** Joreintje D Mackenbach, Elodie L Ibouanga, Monique H van der Veen, Kirsten A Ziesemer, Maria G M Pinho

**Affiliations:** Department of Epidemiology and Data Science, Amsterdam Public Health Research Institute, Amsterdam UMC, Amsterdam, The Netherlands; Upstream Team, Amsterdam UMC, Amsterdam, The Netherlands; Department of Epidemiology and Data Science, Amsterdam Public Health Research Institute, Amsterdam UMC, Amsterdam, The Netherlands; Departments of Preventive Dentistry and Pediatric Dentistry, Academic Centre for Dentistry Amsterdam, University of Amsterdam and VU University Amsterdam, Amsterdam, The Netherlands; Department of Oral hygiene, University for Applied Sciences Inholland, Amsterdam, The Netherlands; Medical Library, Vrije Universiteit, Amsterdam, The Netherlands; Department of Epidemiology and Data Science, Amsterdam Public Health Research Institute, Amsterdam UMC, Amsterdam, The Netherlands; Upstream Team, Amsterdam UMC, Amsterdam, The Netherlands

## Abstract

**Background:**

There is increasing evidence that the food environment, i.e. the availability, accessibility, price and promotion of foods and beverages, has a significant influence on oral health through food consumption. With this systematic literature review, we systematically summarize the available evidence on relations between the food environment and oral health outcomes in children and adults.

**Methods:**

English-language studies were identified through a systematic literature search, executed by a medical information specialist, on OVID/Medline, Embase, Web of Science and CINAHL. Title and abstract screening, full-text screening and quality assessment [using the Quality Assessment with Diverse Studies (QuADS) tool] were done independently by two authors.

**Results:**

Twenty-three studies were included, of which 1 studied the consumer food environment (food labeling), 3 the community food environment (e.g. number of food stores in the community), 5 the organizational food environment (availability of healthy foods and beverages in schools), 2 the information environment (television advertisements) and 13 government and industry policies related to the food environment (e.g. implementation of a sugar-sweetened beverage tax). Almost all studies found that unhealthy food and beverage environments had adverse effects on oral health, and that policies improving the healthiness of food and beverage environments improved—or would improve in case of a modeling study—oral health.

**Conclusions:**

This systematic literature review provides evidence, although of low to moderate quality and available in a low quantity only, that several aspects of the food environment, especially policies affecting the food environment, are associated with oral health outcomes.

## Introduction

Despite being largely preventable, the prevalence and burden of oral diseases has dramatically increased between 1990 and 2015.^1^ Oral diseases are now the most common non-communicable diseases (NCDs) across the globe and there is particular concern over their rising prevalence in low- and middle-income countries (LMICs).^1^ Oral diseases cover a wide range of diseases affecting the soft and hard tissues of the mouth, but key public health priorities include dental caries (tooth decay), periodontal (gum) disease and oral cancer.^2^ Oral diseases are often lifelong conditions that track from childhood into adolescence and adulthood and disproportionally affect vulnerable populations, such as those with low income. Indeed, the prevalence and severity of oral diseases is strongly socioeconomically patterned.^3^

While inadequate exposure to fluoride and poor access to oral health care services contribute to the sustained burden of oral disease, the growing consumption of unhealthy food and beverages is a major contributor to both the prevalence and burden of oral diseases.^2^ In fact, oral diseases share this risk factor, and causality, with a range of other NCDs, such as type 2 diabetes, cardiovascular diseases, chronic respiratory diseases and cancer.^4^ There are several ways in which food and beverage consumption affect oral health, with especially consistent evidence for the cariogenicity of free sugars: sugar acts as a substrate for oral bacteria that produce demineralizing acids.^5^^,^^6^ In addition, the consumption of acidic foods and beverages leads to dental erosion, which can lead to cavities or (dental) hypersensitivity.^7^ The consumption of saturated fatty acids may promote the growth of certain proteolytic bacteria leading to periodontal disease, and alcohol consumption is a major risk factor for oral cancer.^8^ In addition, there is novel evidence that commercial processing methods contribute to the cariogenicity of foods in the mouth, e.g. extrusion-cooking leads to greater acidity of white flour suspensions than regular cooking.^6^ Indeed, tentative evidence in young children shows that higher ultra-processed food and drink (UPFD) consumption is associated with higher risk of caries.^9^ All in all, the oral microbiome plays an important role in the development of oral diseases^10^^,^^11^ and there is evidence that major historical dietary shifts have been accompanied by significant changes in the oral microbiome.^12^

While a biomedical approach is needed for the development and delivery of clinical preventive interventions, the widespread prevalence of oral diseases suggests that population-wide upstream strategies should address the social and commercial determinants of dietary intake and oral health.^2^^,^^13^ It is likely that the availability, accessibility, price and promotion of unhealthy foods and beverages have a significant influence on life course food preferences, purchases and consumption, distortions in the microbiome, infections and inflammation and thus oral health.^2^ Vandevijvere et al.^14^ demonstrated a global increase in the sales of UPFD, which was associated with upward population-level body mass index trajectories. Given that carbonated drinks were the biggest contributor to this global increase in sales on ultra-processed drinks, this may also have contributed to an increase in dental erosion and caries.

**Figure 1:**
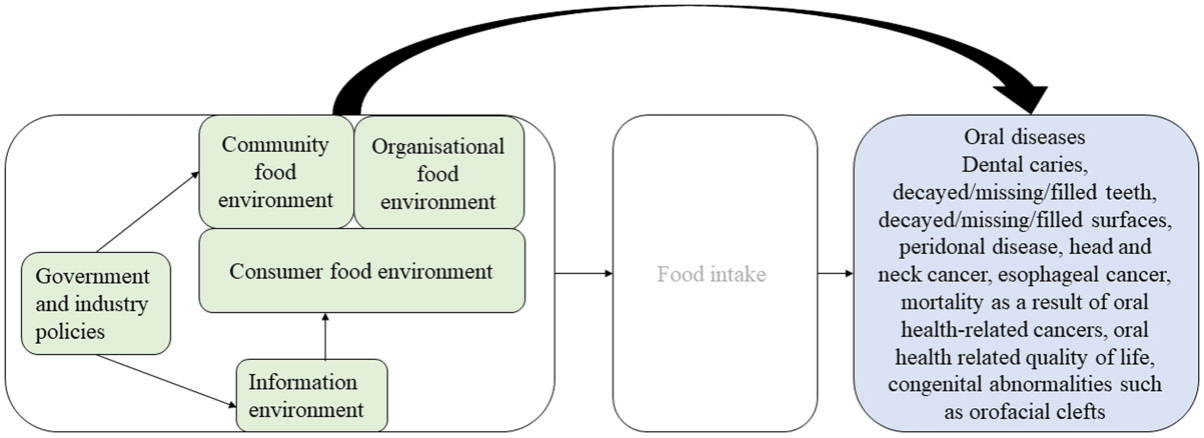
Focus of this systematic review: relation between the food environment and oral health outcomes (footnote: conceptualization of food environment based on Glanz et al.^24^)

There is consistent evidence that the availability,^15^^,^^16^ price,^17^^,^^18^ promotion^19–21^ and nutrition information^15^ of unhealthy foods and beverages in stores, and less consistent evidence that the type, availability and accessibility of food outlets (e.g. Bivoltsis et al.^22^, Caspi et al.^23^) has an influence on dietary intake.^24^ However, to what extent these effects of food environment exposures translate to oral health outcomes is currently unknown. As such, we aimed to systematically summarize the available evidence for the relation between aspects of the food environment and oral health outcomes in children and adults. Figure 1 depicts the association of interest. Our review question was as follows: ‘What is the relation between different aspects of the food environment and oral health outcomes in children and adults?’

## Methods

A systematic literature review of peer-reviewed scientific articles on the relation between aspects of the food environment and oral health outcomes in children and adults was performed. This review is written according to the most recent Preferred Reporting Items for Systematic Reviews and Meta-Analyses (PRISMA) guidelines^25^ and the review protocol was prospectively registered in the PROSPERO International Prospective Register of Systematic Reviews (registration number: CRD42021249013).

The aim was to include primary studies on human subjects, published in English language that addressed the relation between the food environment and oral health outcomes. The food environment was classified according to the ecological model by Glanz et al.^24^ that distinguishes between the community food environment (type and location of food outlets and accessibility of food outlets), the consumer food environment (availability, price, promotion, placement and nutrition information), the organizational food environment (e.g. in schools and workplaces), the information environment (media and advertising) and government and industry policies shaping these food environments. Exclusion criteria were: non-peer-reviewed literature, retracted publications and studies that focused on other aspects of the food environment. We did not apply exclusion criteria regarding the determination of oral health outcomes.

To identify all relevant publications, systematic searches in the bibliographic databases Embase.com, OVID/Medline and Clarivate Analytics/Web of Science Core Collection from inception up to 29 April 2021 were conducted in collaboration with a medical information specialist (K.A.Z.). The following terms, including synonyms and closely related words, were used: ‘food environment’, ‘food supply’, ‘food security’, ‘food marketing’, ‘oral health’, ‘oral neoplasm’ and ‘oral microbiome’. Articles published in other languages than English were not excluded during the search. See Supplementary appendix S1 for the full search strategy. After an initial literature search, two authors (E.L.I. and M.G.M.P.) performed a pilot title and abstract screening test on 100 search results. After subsequent adaptations of the search strings, the medical information specialist (K.A.Z.) performed the final search and deduplicated the search results with EndNote (version X9.3.3) using the Amsterdam Efficient Deduplication method and the Bramer method.^26^ Titles and abstracts, and full-text articles, were screened in duplicate (by E.L.I. and either M.G.M.P. or J.D.M.) and any disagreements about in- or exclusion were discussed among these authors. During this phase, non-English publications were excluded. Rayyan,^27^ a free online application for selecting studies and conducting systematic reviews, was used for both the pilot test and final selection.

After making the final selection, data were extracted (primarily by E.L.I. and checked by J.D.M. and M.G.M.P.) on:

- Study characteristics: name of the first author, year of publication, study design, objective, the country in which the study was conducted and sample size.
- Population characteristics: age, sex, ethnic background.
- Type of food environment: community food environment (type and location of food outlets and accessibility of food outlets), the consumer food environment (availability, price, promotion, placement and nutrition information), the organizational food environment (e.g. in schools and workplaces), the information environment (media and advertising) and government and industry policies shaping these food environments.
- Oral disease reported: dental caries, decayed/missing/filled teeth (DMFT for permanent dentition, dmft for primary dentition), decayed/missing/filled surfaces (DMFS/doffs), periodontal disease, head and neck cancer (HNC), esophageal cancer, mortality as a result of HNC and other oral health-related cancers, oral health-related quality of life (OHRQoL) and congenital abnormalities, such as orofacial clefts.
- Study’s key findings: effect sizes and significance or qualitative summary.
- Study’s conclusion.

Meta-analysis of the results was not possible due to heterogeneous study designs, exposures and outcomes and therefore a narrative synthesis of the results is provided.

Allowing for heterogeneity in study designs, the tool for QuADS was used to assess the quality of included studies.^28^ This tool consists of 13 items and each item receives a score between 0 and 3, with a maximum score of 42 points. Some items were not applicable to some studies, so the maximum score differed between studies. As such, the total score was calculated as a percentage of item scores divided by the total maximum score. As described by Harrison et al.,^28^ the tool does not allow for quantitative cut-offs for high-, medium- or low-quality studies. As such, the quality assessment findings are narratively discussed and the range in quality scores (percentages) per food environment type is presented. The quality assessment was done in duplicate by E.L.I. and J.D.M. and any inconsistencies were discussed with M.G.M.P.

## Results

**Figure 2:**
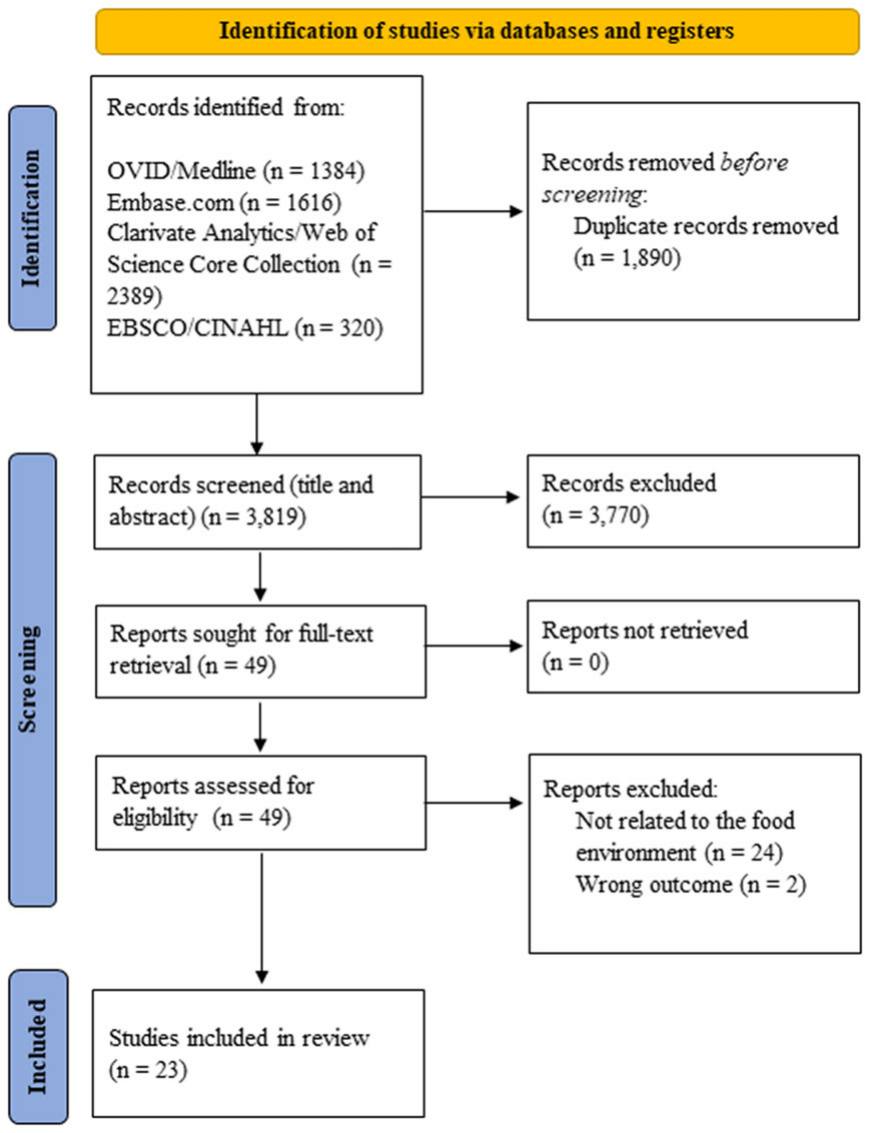
Flowchart showing the study selection according to the PRISMA statement

After deduplication, 3819 articles were left for the title and abstract screening. After exclusion of 3770 articles (of which 125 due to non-English language), 49 articles were eligible for the full-text screening, which led to the inclusion of 23 articles. The other 26 studies were excluded for the following reasons: not related to the food environment (*n* = 24) or wrong outcome (*n* = 2). The process of the study selection is displayed in figure 2.

Due to restrictions in the number of references allowed, we refer to included studies through alphabetical references for which the full list is provided in Supplementary appendix S2.

### Study characteristics

The characteristics of all studies are shown in table 1. Six studies were conducted in USA^a^^–^^f^, three studies were conducted in Thailand^g^^–^^i^ and two studies were conducted in Australia^j,^^k^, Germany^l,^^m^, India^n,^^o^ and North-Ireland^p,^^q^ each. The remaining studies were conducted in Canada^r^, Iraq^s^, Mexico^t^, the Netherlands^u^, New Zealand^v^ and UK^w^ (*n* = 1 each).

**Table 1:** Characteristics of included studies on the food environment and oral health

First author (year)	Country	Study design	Objective	Sample	Type of food environment	Food environment exposure measure(s)	Oral health outcome(s)	Key finding(s)
Alattas et al. (2020)^a^	USA	Observational: cross-sectional	Assess the effects of alcohol control policies on the national mortality rates due to cancers over a 5-year period	US population	Government and industry policies	Restrictiveness of state-level alcohol control policies	Esophageal, laryngeal and oropharyngeal cancer mortality rate	More restrictive state-level alcohol policies were associated with lower mortality rates due to esophageal cancer [−0.06 (95% CI −0.16, 0.04)], laryngeal cancer [−0.03 (95% CI −0.07, 0.01)] and oropharyngeal cancer [−0.04; (95% CI −0.07, −0.01)]
Briggs et al. (2017)^w^	UK	Health impact assessment	Evaluate the oral health impact of a nationwide tiered law that targets large SSB producers and aims to reduce sugar intake	2014 UK population	Government and industry policies	Six scenarios of sugar reduction due to reformulation, price change or change to the SSB market share following legislation for a soft drinks levy	Dental caries	The optimal reformulation scenario could lead to a reduction of 269 375 (82 211–470 928) decayed, missing or filled teeth per year while the least optimal change in market share scenario could lead to an increase of 16 401 (4604–28 037) decayed, missing or filled teeth per year. Five out of six scenarios showed reductions in the number of decayed, missing or filled teeth per year
Choi et al. (2021)^b^	USA	Modeling study	Explore how decreased access to sugary drinks in a national food assistance program affects oral health outcomes over a 10-year period	10 000 Americans aged 2–19	Government and industry policies	Replacing sugary drinks with milk and 100% fruit juice	Dental caries	The replacement of sugary drinks is expected to lead to a decrease in the individual dental caries experience by 0.53 (95% CI −0.55 to −0.51)
Edasseri et al. (2017)^r^	Canada	Cohort	Explore how children’s oral health is affected by school environments and neighborhood environmental factors over a 3-year period	330 white children in Quebec aged 8–10 years	Organizational food environment and community food environment	Healthy eating promotion policies in schools and the schools’ proximity to convenience stores/fast-food restaurants	Dental caries	Children in school types with a >500 m distance from convenience stores/fast-food restaurants and/or better healthy eating promotion policies were found to have a 21% [IRR 0.79 (95% CI 0.68, 0.90)] and 6% [IRR 0.94 (95% CI 0.83, 1.07)] lower dental caries experience than children in schools within 500 m from convenience stores/fast-food restaurants and worse eating promotion policies
Freeman and Oliver (2009)^q^	North-Ireland	Randomized controlled trial	Evaluate the effects of a school-based food policy that aims to improve oral health outcomes in school children 2 years after its implementation	345 9-year olds from 16 schools	Organizational food environment	Boosting Better Breaks (BBB): a policy that increases the availability of healthy food and decreases access to unhealthy food in schools	Obvious tooth decay and decay in dentin	After a 2-year follow-up, obvious tooth decay had increased overall, and there was no effect of the school food intervention. Decay into dentin at follow-up was predicted by intervention status of the school (*P* < 0.05). The results may be explained by increased out-of-school snack consumption
Freeman et al. (2001)^p^	North-Ireland	Randomized controlled trial	Assess the 2-year impact of a school-based policy that aims to facilitate healthy food habits on oral health outcomes	364 9-year old school children from 16 schools	Organizational food environment	Boosting Better Breaks (BBB): a policy that increases the availability of healthy food and decreases access to unhealthy food in schools	Dental caries	The number of children without caries in BBB schools decreased from 33% after 1 year to 27% after 2 years. The increase in DMFT between both years was significant (F[1.85]=22.01; *P* < 0.001)
Ghimire and Rao (2013)^n^	India	Cross-sectional	Estimate the extent to which oral health outcomes are associated with exposure to television advertisements	600 school children	Information food environment	Television advertisements of food and beverages	Dental caries	Children who watched advertisements and asked their parents for the advertised food and beverages had significantly more dental caries
Hernández et al. (2021)^t^	Mexico	Natural experiment	Evaluate how an additional nationwide food and beverage tax has affected the quarterly dental caries experience	2 648 893 Mexicans aged 1–99 years	Government and industry policies	Taxes on SSBs and unhealthy food	Dental caries	The number of people with dental caries decreased with 107.5 in dmft and 393.6 in DMFT. Dental caries experience decreased with 0.004 dmft and 0.026 DMFT every 3 months
Jamel et al. (2004)^s^	Iraq	Prospective cohort	Compare oral health outcomes in school children of different areas before and after the implementation of a sanction that restricted food availability in the country	3015 school children aged 6–7, 11–12 and 14–15 from one urban and seven rural areas	Government and industry policies	United Nations Sanction (UNS) that limited sugar availability	Dental caries	The number of caries-free children increased from 16.4% to 34.6% in the urban sample and from 61.4% to 88.4% in the rural sample after the implementation of the sanction. This change happened in the span of 5 years
Jevdjevic et al. (2019)^u^	The Netherlands	Modeling study	Estimate how a nationwide SSB tax may affect oral health outcomes in children and adults over a lifetime	6- to 79-year olds	Government and industry policies	20% SSB tax	Dental caries	The tax is expected to avert 1 030 163 (95% UI 1 027 903–1 032 423) caries lesions, which is an individual gain of 2.13 (95% UI 2.12–2.13) caries-free tooth years over a lifetime
Jevdjevic et al. (2021)^m^	Germany	Modeling study	Estimate how a food labeling system may affect oral health outcomes in children and adults when implemented throughout the country	500 000 Germans aged 14–79 in 2017	Consumer food environment	Front-of-package food labeling	Dental caries and burden of disease	The expected effect of the food labeling system is the aversion of 2 370 715 (95% CI 2 062 730–2 678 700) caries lesions and an increase in the OHRQoL as a result of the prevention of 677.62 (95% CI, 589.59–765.65) DALYs over a 10-year period
Jiang et al. (2019)^k^	Australia	Retrospective cohort	Explore the gender-based differences in the impact of alcohol control policies on HNC mortality rates throughout >50 years	Australians aged 15+ years	Government and industry policies	Alcohol control policies	HNC mortality rates	Liquor license liberalization was associated with a significant increase in HNC mortality in both men [0.11 (95% CI 0.03; 0.20)] and women [0.04 (95% CI 0.02; 0.06)]
Kaewkamnerdpong and Krisdapong (2018)^i^	Thailand	Cross-sectional	Evaluate the oral health outcomes of a school food program that aims to decrease sugar intake among 55 schools	984 children from 55 schools	Organizational food environment	Provision of fruits with school meals and sale of healthy non-sugary snacks	Dental caries	Students of schools that included fresh fruits with school meals and sold healthy non-sugary snacks were found to have a significantly lower number of tooth decay and DMFT than students of schools that sold sugary snacks and beverages and did not provide fruits with school meals [aOR 0.99 (95% CI 0.64; 1.54)]
Maupomé et al. (2010)^e^	USA	Ecological	Estimate the effects of a community-based intervention that aims to prevent ECC in an 18–30 months period	178 American Indian infants from three communities	Community food environment	Increased water availability	ECC	The intervention was associated with a significant decrease in early-stage (between 0.300 and 0.631) and advanced-stage carious lesions (between 0.342 and 0.449). The separate effects of water consumption promotion were not reported
Schwendicke et al. (2016)^l^	Germany	Modeling study	Estimate the health effects of the implementation of a nationwide SSB tax over a 10-year period	Germans aged 14–79 in 2015	Government and industry policies	20% SSB tax	Dental caries	The tax is expected to lead to 750 000 fewer new caries lesions compared to a 0% tax
Somasundaram et al. (2018)^o^	India	Cross-sectional	Estimate the extent to which oral health outcomes are associated with exposure to television advertisements	300 children from one school, aged 6–8 years	Information food environment	Television advertisements	Dental caries	A higher DMFS was found in children who asked for advertised food and soft drinks than children who did not. The effects were not quantified
Sowa et al. (2019)^j^	Australia	Modeling study	Estimate the health effects of the implementation of a nationwide SSB tax over a 10-year period	Australians aged 6 years and up	Government and industry policies	SSB tax	Dental caries	The tax is estimated to lead to the prevention of 3.9 million DMFT [95% CI (6.08–1.73 million)]
Tellez et al. (2006)^f^	USA	Cross-sectional	Investigate how environmental factors affect oral health outcomes in caregivers of low-income children	Caregivers of 1021 low-income African American children in Detroit	Community food environment	Number of grocery stores in the neighborhood	Dental caries	A significant association was found between the number of grocery stores in a neighborhood and dental caries severity. Caregivers in neighborhoods with a high number of grocery stores had a higher caries experience than caregivers in neighborhoods with a lower number of grocery stores (2.1 *P* < 0.05)
Thornley et al. (2017)^v^	New Zealand	Non-randomized controlled trial	Evaluate the differences in oral health outcomes of a policy that aims to reduce sugar consumption in school children over a 7-year period	3813 children from 10 schools	Organizational food environment	Ban on sugary drinks, ‘water only’ policy and provision of water bottles on school grounds	Dental caries	Children exposed to the policy experienced less dental caries [0.37 (95% CI: −0.092; −0.652)] than children of other schools who were not exposed to the policy
Urwannachotima et al. (2019)^g^	Thailand	Qualitative case study	Describe the association between the implementation of a nationwide SSB tax and oral health outcomes	Thai population	Government and industry policies	SSB tax	Dental caries	A CLD was developed to describe the different ways in which the tax may impact oral health. It was concluded that the tax needs to be combined with other interventions to improve oral health outcomes
Urwannachotima et al. (2020)^h^	Thailand	Modeling study	Identify the potential health effects of a national SSB tax over a 30-year period	Thai population aged 15 years and older	Government and industry policies	SSB tax	Dental caries	Without the tax, the dental caries prevalence is expected to increase by 13.6% compared to 1% with the tax. Under the aggressive scenario the prevalence is expected to decrease by 21%
Yang et al. (2016)^c^	USA	Retrospective cohort	Explore effects of the nationwide folic acid fortification of grains on the statewide prevalence of orofacial clefts	1 366 369 children born in central California between 1989 and 2010	Government and industry policies	Mandatory folic acid fortification	Orofacial clefts	The prevalence of CLP increased by 0.2 per 100 000 births (95% CI −6.3; 6.6) before the fortification and decreased by 2.1 (95% CI −3.9; −0.3) after fortification. For CP, the pre fortification increase was 1.2 (95% CI −5.3; 7.7), the prevalence decreased by 2.3 (95% CI −4.7; 0.01) after fortification
Yazdy et al. (2007)^d^	USA	Retrospective cohort	Explore effects of the nationwide folic acid fortification of grains on the national prevalence of orofacial clefts	45 926 598 children born between 1990 and 2002	Government and industry policies	Mandatory folic acid fortification	Orofacial clefts	The prevalence of orofacial clefts decreased by 5.0/100 000 births [PR = 0.94 (95% CI: 0.92–0.96)] after the fortification

AOR, adjusted odds ratio; 95% CI, 95% confidence interval; CLD, causal loop diagram; CLP, cleft lip with/without palate; CP, cleft palate; dmft, decayed/missed/filled teeth in the primary dentition; DMFT, decayed/missed/filled teeth in the permanent dentition; ECC, early childhood caries; HNC, head and neck cancer; IRR, incidence rate ratio; PR, prevalence ratio; SSB, sugar-sweetened beverage; UI, uncertainty interval; UK, United Kingdom; US, United States.

Out of 23 studies, 19 used dental caries (tooth decay) as outcome^b,^^e–j,^^l^^–^^w^, 2 used oral or HNC outcomes^a,^^k^ and 2 focused on the prevalence of orofacial clefts^c,^^d^. Six were cross-sectional observational studies^a,^^f,^^i^^,^^n,^^o,^^r^, four were longitudinal observational studies^c,^^d,^^k,^^t^, five were experimental studies^e,^^p,^^q,^^t,^^v^, six were modeling studies^b,^^h,^^j,^^l,^^m,^^u^, one was a qualitative study^g^ and one was a health impact assessment^w^.

### Narrative synthesis

Only one study investigated the ‘consumer food environment’, with a focus on food labeling^m^. This was a modeling study that showed that the implementation of front-of-pack food labeling could potentially lead to the aversion of 2 370 715 (95% CI: 2 062 730–2 678 700) cases of caries and 677.62 (95% CI: 589.59–765.65) daily life-years lost over a 10-year period.

Three studies investigated the ‘community food environment’^e,^^f, r^, of which two studied geographic access to food stores^f,^^r^ and one the modification of drinking environments (increasing water availability)^e^. Edassiri et al. found that Canadian children at schools located >500 m from convenience stores/fast-food restaurants had less dental caries^r^ and Tellez et al. showed that US adults with a higher number of grocery stores in their neighborhood had more dental caries^f^. A US community intervention that also focused on drinking water found significant beneficial intervention effects on early-stage and advanced-stage carious lesions^e^.

All the five studies investigating the ‘organizational food environment’ focused on food and beverage availability in schools^i,^^p^^–^^r,^^v^. Three of them found that healthy school food policies were associated with less caries^i,^^r,^^v^, but two others in North-Ireland showed that tooth decay, caries and DMFT increased after the implementation of a healthy school food policy^p,^^q^.

Two studies investigated the ‘information environment’, both with a focus on television advertisement^n,^^o^. Both studies showed that Indian children who watched food and beverage advertising on television and asked their parents for these foods had more dental caries and a higher DMFS^n,^^o^.

A total of 13 studies investigated the relation between ‘government and industry policies’ and oral health^a^^–^^d,^^g,^^h,^^j^^–^^l,^^s^^–^^u,^^w^, of which 2 focused on alcohol-related policies^a,^^k^, 7 focused on the effects of a sugar-sweetened beverage tax^g,^^h,^^j,^^l,^^t,^^u,^^w^, 2 focused on policies related to availability^b,^^s^ and 2 on folic acid fortification^c,^^d^. The two alcohol-related policy studies showed that more restrictive alcohol policies were associated with lower mortality rates due to esophageal, laryngeal and oropharyngeal cancer^a^ and liberalization of alcohol policies was associated with significant increases in HNC mortality^k^. The seven studies from various countries on sugar-sweetened beverage taxation consistently showed that its implementation would have^g,^^h,^^j,^^l,^^u,^^w^ or has^t^ a positive effect on dental caries, caries lesions, DMFT and the number of decayed, missing or filled teeth per year. The two studies focused on availability-policies demonstrated that restricting unhealthy foods and beverages leads to decreases in caries^b,^^s^, and the two studies evaluating the nationwide folic acid fortification of grains found that the prevalence of orofacial clefts decreased after the fortification^c,^^d^.

### Quality assessment

The quality assessment of included studies is presented in table 2. The quality of the studies was variable (ranging from 18% to 79%), with many studies providing no or a very superficial theoretical underpinning to the research and none but one considered stakeholders in the design or conduct of the study. Lower quality assessment was generally related to a lack of reporting around the rationale for data collection tools, the data collection procedure, information on recruitment data and justification for analytical methods. Given the small number of studies that focused on each type of food environment, it is difficult to qualitatively ‘weigh’ the evidence by the studies’ quality assessments. The only study that investigated the ‘consumer food environment’ was of moderate quality (64%), and the three studies that investigated the ‘community food environment’ (56–79%) and the five studies investigating the ‘organizational food environment’ (54–79%) were also of moderate quality. The two studies investigating the ‘information environment’ were both of low quality (18–33%) so the results from these studies should be interpreted with caution. Most of the 13 studies investigated the relation between ‘government and industry policies’ were of moderate quality (55–74%), with one exception of a study that focused on policies related to availability (41%).

**Table 2:** Quality assessment of included studies according to the QuADS

First author (year)	Type of food environment	Criteria													
		Theoretical or conceptual under pinning to the research	Research aims	Research setting and target popula tion	Appro priate study design	Appro priate sampling	Rationale for data (collection)	Appro priate tools	Data collection procedure	Recruit ment data provided	Justifica tion analytical methods	Appro priate analyses	Stakeholders considered in design or conduct	Strengths and limitations discussed	Total score (%)
Alattas et al. (2020)^a^	Government and industry policies	1	3	2	1	N/A	1	3	2	N/A	3	2	0	3	67
Briggs et al. (2017)^w^	Government and industry policies	0	3	2	3	N/A	1	3	3	N/A	2	2	0	3	66
Choi et al. (2021)^b^	Government and industry policies	0	3	2	3	N/A	1	0	2	N/A	3	3	0	3	61
Edasseri et al. (2017)^r^	Organizational food environment and community food environment	1	3	3	3	1	2	2	1	2	2	3	0	3	66
Freeman and Oliver (2009)^q^	Organizational food environment	0	3	3	3	3	3	3	2	3	2	3	0	2	77
Freeman et al. (2001)^p^	Organizational food environment	0	1	3	3	3	2	3	2	3	0	1	1	3	64
Ghimire and Rao (2013)^n^	Information food environment	1	1	1	1	1	0	0	1	0	0	1	0	0	18
Hernández et al. (2021)^t^	Government and industry policies	1	2	2	3	3	2	2	2	3	3	3	0	3	74
Jamel et al. (2004)^s^	Government and industry policies	0	3	2	2	1	1	3	2	1	0	1	0	0	41
Jevdjevic et al. (2019)^u^	Government and industry policies	0	3	3	3	N/A	1	3	2	N/A	2	3	0	3	70
Jevdjevic et al. (2021)^m^	Consumer food environment	2	2	2	3	N/A	1	2	1	N/A	2	3	0	3	64
Jiang et al. (2019)^k^	Government and industry policies	0	3	2	3	N/A	2	2	2	N/A	3	3	0	3	70
Kaewkamnerdpong and Krisdapong (2018)^i^	Organizational food environment	1	3	3	2	3	1	2	2	3	3	2	0	2	69
Maupomé et al. (2010)^e^	Community food environment	0	2	2	2	1	2	3	2	0	3	3	0	2	56
Schwendicke et al. (2016)^l^	Government and industry policies	0	3	3	2	2/N/A	1	2	2	N/A	2	2	0	3	61
Somasundaram et al. (2018)^o^	Information food environment	1	2	3	1	1	1	1	1	0	1	1	0	0	33
Sowa et al. (2019)^j^	Government and industry policies	2	3	1	1	2/N/A	2	2	2	N/A	3	2	0	0	55
Tellez et al. (2006)^f^	Community food environment	3	3	3	2	3	2	2	2	2	3	3	0	3	79
Thornley et al. (2017)^v^	Organizational food environment	0	3	2	2	1	1	3	2	1	3	1	0	2	54
Urwannachotima et al. (2019)^g^	Government and industry policies	1	3	2	3	2	2	3	3	1	N/A	N/A	3	1	73
Urwannachotima et al. (2020)^h^	Government and industry policies	2	3	1	3	2/N/A	1	2	2	N/A	3	3	3	1	73
Yang et al. (2016)^c^	Government and industry policies	0	2	2	2	2	2	3	3	1	2	2	0	3	62
Yazdy et al. (2007)^d^	Government and industry policies	0	3	2	3	1	2	3	2	1	2	3	0	3	64

## Discussion

This systematic overview of the available evidence for an association between the food environment and oral health outcomes is an important addition to the existing evidence that has shown links between the food environment and dietary intake,^15–24^ and between dietary intake and oral health.^5^^,^^6^^,^^8^^,^^9^ The evidence obtained in this review, although of low to moderate quality and available in a low quantity only, suggests that several aspects of the food environment are associated with oral health outcomes. Most evidence concerned government and industry policies targeting the food environment, which demonstrated that policies restricting access to or increasing prices of unhealthy foods and beverages leads to better oral health, or could lead to better oral health as shown in modeling studies. Evidence for the influence of consumer, community and organizational food environments and the information environment on oral health outcomes was scarce. Yet, with the exception of studies focused on school food policies, this evidence consistently showed that healthier food environments, or making food environments healthier, were associated with better oral health outcomes. However, it should be noted that the quality of both studies on the information environment was low.

The results from this review are in line with a systematic review demonstrating that food insecurity status, whereby families often resort to cheaper and often unhealthier foods, is associated with worse oral health.^29^ Although the relatively small number of included studies did not allow for subgroup analyses, families with lower socioeconomic position may be especially vulnerable to unhealthy food environments; they may lack the energy, time, money, transport and knowledge needed to find affordable and healthy options. Given the chronic and cumulative nature of oral diseases, and their interrelation with chronic diseases, such as type 2 diabetes,^4^ it is also worrying to observe the adverse effects of unhealthy food environments on oral health outcomes in children. Children are very susceptible to marketing strategies that promote unhealthy foods and beverages, such as collectibles, celebrity endorsers or game elements,^30^ but may also form healthy habits early on in life, e.g. when drinking water is easily available.^31^

The evidence from this review is especially important in light of the rapid nutritional transition that is currently being observed in many LMICs^32^ where both the consumption of sugar(ry drinks) and caries prevalence are on the rise.^1^^,^^33^ As a response to increasing regulation of commercial food and beverage companies in high-income countries and the rapid economic growth of LMICs, transnational food and beverage corporations are expanding their market to LMICs (e.g. Baker et al.^34^) thereby putting these communities at risk of diet-related chronic diseases including oral diseases.

The findings from this review imply that the food environment should be taken into account when investigating the determinants of oral health outcomes. Similarly, studies on dietary or oral health interventions should measure the healthiness of participants’ food environments, as the interventions may be differentially effective dependent on how supportive the food environment is for healthy choices. In this review, we only focused on the direct ways that food and beverage industry has on oral health, namely through the availability, promotion and prices of foods and beverages. However, conceptualizations of the commercial determinants of dietary behaviors and obesity also point to more indirect ways of influencing dietary behaviors.^2^^,^^35^ These strategies in the political and legal spheres include framing the evidence and debate, such as in the media or public consultations^36^ influencing the policymaking process, such as lobbying against a sugar tax,^37^ and limiting corporate liability, such as keeping prices low so that they do not reflect the true costs of the damage caused by consumption.^38^ Future studies and systematic reviews could substantiate these conceptualizations with empirical evidence to further strengthen the evidence base on the commercial determinants of oral health.

Our review also has implications for practice. Due to the scarcity of studies, we are unable to conclude whether some aspects of the food environment have a stronger influence on oral health outcomes than others. However, given that government and industry policies can affect community, consumer and organizational food environments, and their information environment, it seems imperative to use policy levers to make healthy food and beverage choices available, attractive and affordable. Indeed, the articles included in this review suggest that nationwide food labeling or sugar taxes could substantially reduce caries, caries-related morbidity and economic burden.^39^^,^^40^ Of course, the availability and promotion of unhealthy foods and beverages in the food environment is beyond the sphere of influence of health care professionals. No clinical treatment can compete with the availability of, marketing for and low prices of unhealthy foods and beverages that are detrimental for oral health. The prevention of diet-related oral diseases therefore requires more upstream interventions and policies targeting the commercial determinants of oral health, especially to protect those in vulnerable socioeconomic conditions.

### Strengths and limitations

Strengths of this systematic literature review include the thorough search in three broad databases conducted by a medical information specialist, considering multiple types of food environments and a range of potentially relevant oral health outcomes. We did not set language restrictions for the search to avoid language publication bias, but had to exclude 3% of abstract during the screening of titles and abstracts due to non-English language. By including a range of study designs, we systematically reviewed the complete evidence base. However, the combination of heterogeneity in study designs and a limited number of studies of each study design hindered the drawing of general conclusions. Related to this, we chose a generic quality assessment tool that had recently been improved,^28^ which was suitable for all types of study designs. The limitation of this tool is that some aspects that are clearly important indicators of quality for specific study designs, e.g. addressing confounding bias for observational studies, were not assessed. However, with the observed heterogeneity in study designs it would not have been feasible to assess each of them with separate tools, and this would also have resulted in limited comparability of quality across study designs.

## Conclusions

Although the number of available studies is limited, and their quality is variable, this systematic literature review provides evidence that several aspects of the food environment are associated with oral health outcomes. Most available evidence was from studies that modeled government and industry policies targeting the food environment. Such studies demonstrated that policies restricting access to or increasing prices of unhealthy foods and beverages could lead to better oral health.

## Supplementary data

Supplementary data are available at *EURPUB* online.

## Funding

No specific funding was ascertained to write this systematic review.

*Conflicts of interest*: None declared.

Key points

- An increasing number of food environment studies focus on oral health outcomes.
- We systematically summarized the available evidence.
- We studied aspects of the consumer, community and organizational food environment, information environment and government and industry policies.
- There is lack of good quality studies, but evidence consistently points toward adverse effects of unhealthy food environments on oral health outcomes.
- The adverse effects of unhealthy food environments should be considered in the consideration of implementing food policies.

## Supplementary Material

ckac086_Supplementary_Data
