# Supplementary material for: Relation between the food environment and oral health—systematic review
Source: Eur J Public Health. 2022 Jul 18;32(4):606–16. doi: 10.1093/eurpub/ckac086 (PMC9341680; doi:10.1093/eurpub/ckac086)
Supplement: ckac086_Supplementary_Data [file ckac086_supplementary_data.docx]

# Appendix 1

OVID/Medline 1384 results (29 April 2021)

The searches for all databases can be found at doi:10.7302/z2vh5m1h.

| **Search** | **Medline Query – April 29, 2021** | **Results** |
| --- | --- | --- |
| 1 | exp Food Supply/ or exp Food labeling/ or exp Legislation, Food/ or ((food or drink or beverage or alcohol or nutritional or dietary) adj3 (label* or legislat* or environment* or access* or availab* or adverti* or market* or promot* or policy or price* or afford* or sponsor* or cost* or supply or tax* or retail or outlet or expos* or packag* or security or insecurity or regulat* or divers* or rules or spatial-access or Product-placement)).ti,ab,kf. or (foodscap* or food-desert).ti,ab,kf. | 107129 |
| 2 | exp Oral Health/ or exp Mouth Diseases/ or exp Tooth Diseases/ or ((oral or mouth or tooth or dental or gingiv* or gum or periodont*) adj3 (health or disease* or ulcer or function or hygiene or quality-of-life or status)).ti,ab,kf. or ((caries or cariogenesis or carious or cavity or plaque or debris or decay or erosion or Dmft or Dmfs or loss or fluorosis) adj3 (fissure or dental or dentin* or tooth or teeth or enamel or molar* or root)).ti,ab,kf. or (malocclusion or white spot lesion or edentation or periodontitis or masticatory-performance or masticatory-function or chew* or swallow or halitos* or xerostomia or pulpitis).ti,ab,kf. | 541457 |
| 3 | (adenoma* or anticarcinogen* or blastoma* or cancer* or carcinogen* or carcinom* or carcinosarcoma* or chordoma* or malignan* or melanom* or mesenchymoma* or metasta* or neoplas* or neuroma* or nsclc or oncogen* or oncolog* or paraneoplastic or plasmacytoma* or precancerous or sarcoma* or teratocarcinoma* or teratoma* or tumor* or tumour*).ti,ab,kf. | 3773882 |
| 4 | exp Neoplasms/ | 3451445 |
| 5 | (face or facial* or gingiva* or head or hypopharyn* or jaw or jaws or laryn* or lip or lips or mandib* or mouth or nasopharyn* or neck or nose or oral* or oropharyn* or otorhinolaryn* or palatal* or palate or palatum or paranasal* or paroti* or pharyn* or salivar* or sublingual* or submandib* or throat or tongue or tonsil*).ti,ab,kf. | 1946705 |
| 6 | exp Mouth Neoplasms/ or ((3 or 4) and 5) | 416810 |
| 7 | ((oral or mouth or tooth or dental or gingiv* or gum or periodont*) adj3 (microbio* or flora or microflora or bacteri* or microb* or microorganism* or micro-organism*)).ti,ab,kf. | 17874 |
| 8 | 1 and (2 or 6 or 7) | 1699 |
| 9 | 8 not (exp Animals/ not exp Humans/) | 1432 |
| 10 | 9 not review.ti. | 1384 |

**Embase.com 1616 results (29 April 2021)**

| **Search** | **Embase Query – April 29, 2021** | **Results** |
| --- | --- | --- |
| #8 | #7 NOT 'review':ti | 1616 |
| #7 | #6 NOT ([animals]/lim NOT [humans]/lim) | 1676 |
| #6 | #5 NOT 'conference abstract'/it | 2040 |
| #5 | #1 AND (#2 OR #3 OR #4) | 2464 |
| #4 | 'oral microbiome'/exp OR 'mouth flora'/exp OR ((('oral' OR 'mouth' OR 'tooth' OR 'dental' OR 'gingiv*' OR 'gum' OR 'periodont*') NEAR/3 ('microbio*' OR 'flora' OR 'microflora' OR 'bacteri*' OR 'microb*' OR 'microorganism*' OR 'micro-organism*')):ti,ab,kw) | 24850 |
| #3 | 'mouth tumor'/exp OR (('neoplasm'/exp OR 'adenoma*':ti,ab,kw OR 'anticarcinogen*':ti,ab,kw OR 'blastoma*':ti,ab,kw OR 'cancer*':ti,ab,kw OR 'carcinogen*':ti,ab,kw OR 'carcinom*':ti,ab,kw OR 'carcinosarcoma*':ti,ab,kw OR 'chordoma*':ti,ab,kw OR 'malignan*':ti,ab,kw OR 'melanom*':ti,ab,kw OR 'mesenchymoma*':ti,ab,kw OR 'metasta*':ti,ab,kw OR 'neoplas*':ti,ab,kw OR 'neuroma*':ti,ab,kw OR 'nsclc':ti,ab,kw OR 'oncogen*':ti,ab,kw OR 'oncolog*':ti,ab,kw OR 'paraneoplastic':ti,ab,kw OR 'plasmacytoma*':ti,ab,kw OR 'precancerous':ti,ab,kw OR 'sarcoma*':ti,ab,kw OR 'teratocarcinoma*':ti,ab,kw OR 'teratoma*':ti,ab,kw OR 'tumor*':ti,ab,kw OR 'tumour*':ti,ab,kw) AND ('face':ti,ab,kw OR 'facial*':ti,ab,kw OR 'gingiva*':ti,ab,kw OR 'head':ti,ab,kw OR 'hypopharyn*':ti,ab,kw OR 'jaw':ti,ab,kw OR 'jaws':ti,ab,kw OR 'laryn*':ti,ab,kw OR 'lip':ti,ab,kw OR 'lips':ti,ab,kw OR 'mandib*':ti,ab,kw OR 'mouth':ti,ab,kw OR 'nasopharyn*':ti,ab,kw OR 'neck':ti,ab,kw OR 'nose':ti,ab,kw OR 'oral*':ti,ab,kw OR 'oropharyn*':ti,ab,kw OR 'otorhinolaryn*':ti,ab,kw OR 'palatal*':ti,ab,kw OR 'palate':ti,ab,kw OR 'palatum':ti,ab,kw OR 'paranasal*':ti,ab,kw OR 'paroti*':ti,ab,kw OR 'pharyn*':ti,ab,kw OR 'salivar*':ti,ab,kw OR 'sublingual*':ti,ab,kw OR 'submandib*':ti,ab,kw OR 'throat':ti,ab,kw OR 'tongue':ti,ab,kw OR 'tonsil*':ti,ab,kw)) | 607841 |
| #2 | 'dental health'/exp OR 'mouth disease'/exp OR ((('oral' OR 'mouth' OR 'tooth' OR 'dental' OR 'gingiv*' OR 'gum' OR 'periodont*') NEAR/3 ('health' OR 'disease*' OR 'ulcer' OR 'function' OR 'hygiene' OR 'quality of life' OR 'status' OR 'bleeding' OR 'hemorr*' OR 'haemorr*')):ti,ab,kw) OR ((('caries' OR 'cariogenesis' OR 'carious' OR 'cavity' OR 'plaque' OR 'debris' OR 'decay' OR 'erosion' OR 'dmft' OR 'dmfs' OR 'loss' OR 'fluorosis') NEAR/3 ('fissure' OR 'dental' OR 'dentin*' OR 'tooth' OR 'teeth' OR 'enamel' OR 'molar*' OR 'root')):ti,ab,kw) OR 'malocclusion':ti,ab,kw OR 'white spot lesion':ti,ab,kw OR 'edentation':ti,ab,kw OR 'periodontitis':ti,ab,kw OR 'masticatory performance':ti,ab,kw OR 'masticatory function':ti,ab,kw OR 'chew*':ti,ab,kw OR 'swallow':ti,ab,kw OR 'halitos*':ti,ab,kw OR 'xerostomia':ti,ab,kw OR 'pulpitis':ti,ab,kw OR 'noma':ti,ab,kw OR 'oral thrush':ti,ab,kw OR 'early childhood caries':ti,ab,kw OR 'baby bottle syndrome':ti,ab,kw OR 'nursing caries':ti,ab,kw | 738724 |
| #1 | 'food environment'/exp OR 'food legislation'/exp OR 'food access'/exp OR 'food advertising'/exp OR 'food packaging'/exp OR ((('food*' OR 'drink*' OR 'beverage*' OR 'alcohol' OR 'nutrition*' OR 'diet*') NEAR/3 ('label*' OR 'legislat*' OR 'environment*' OR 'access*' OR 'availab*' OR 'adverti*' OR 'market*' OR 'promot*' OR 'policy' OR 'price*' OR 'afford*' OR 'sponsor*' OR 'cost*' OR 'supply' OR 'tax*' OR 'retail' OR 'outlet' OR 'packag*' OR 'security' OR 'insecurity' OR 'regulat*' OR 'divers*' OR 'rules' OR 'spatial access' OR 'product placement')):ti,ab,kw) OR 'foodscap*':ti,ab,kw OR 'food desert':ti,ab,kw | 132512 |

**Clarivate Analytics/Web of Science Core collection (29 April 2021)**

| **Search** | **Web of Science Query – April 29, 2021** | **Results** |
| --- | --- | --- |
| #6 | #5 NOT TI=(“review”) | 2,389 |
|  | *Indexes=SCI-EXPANDED, SSCI, A&HCI, ESCI Timespan=All years* |  |
| #5 | #1 AND (#2 OR #3 OR #4) | 2,482 |
|  | *Indexes=SCI-EXPANDED, SSCI, A&HCI, ESCI Timespan=All years* |  |
| #4 | TS=((“oral” OR “mouth” OR “tooth” OR “dental” OR “gingiv*” OR “gum” OR “periodont*”) NEAR/3 (“microbio*” OR “flora” OR “microflora” OR “bacteri*” OR “microb*” OR “microorganism*” OR “micro-organism*”) ) | 19,884 |
|  | *Indexes=SCI-EXPANDED, SSCI, A&HCI, ESCI Timespan=All years* |  |
| #3 | TS=((“adenoma*” OR “anticarcinogen*” OR “blastoma*” OR “cancer*” OR “carcinogen*” OR “carcinom*” OR “carcinosarcoma*” OR “chordoma*” OR “malignan*” OR “melanom*” OR “mesenchymoma*” OR “metasta*” OR “neoplas*” OR “neuroma*” OR “nsclc” OR “oncogen*” OR “oncolog*” OR “paraneoplastic” OR “plasmacytoma*” OR “precancerous” OR “sarcoma*” OR “teratocarcinoma*” OR “teratoma*” OR “tumor*” OR “tumour*”) AND (“face” OR “facial*” OR “gingiva*” OR “head” OR “hypopharyn*” OR “jaw” OR “jaws” OR “laryn*” OR “lip” OR “lips” OR “mandib*” OR “mouth” OR “nasopharyn*” OR “neck” OR “nose” OR “oral*” OR “oropharyn*” OR “otorhinolaryn*” OR “palatal*” OR “palate” OR “palatum” OR “paranasal*” OR “paroti*” OR “pharyn*” OR “salivar*” OR “sublingual*” OR “submandib*” OR “throat” OR “tongue” OR “tonsil*”) ) | 364,975 |
|  | *Indexes=SCI-EXPANDED, SSCI, A&HCI, ESCI Timespan=All years* |  |
| #2 | TS= (((“oral” OR “mouth” OR “tooth” OR “dental” OR “gingiv*” OR “gum” OR “periodont*”) NEAR/3 (“health” OR “disease*” OR “ulcer” OR “function” OR “hygiene” OR “quality of life” OR “status”) ) OR ((“caries” OR “cariogenesis” OR “carious” OR “cavity” OR “plaque” OR “debris” OR “decay” OR “erosion” OR “Dmft” OR “Dmfs” OR “loss” OR “fluorosis”) NEAR/3 (“fissure” OR “dental” OR “dentin*” OR “tooth” OR “teeth” OR “enamel” OR “molar*” OR “root”) ) OR (“malocclusion” OR “white spot lesion” OR “edentation” OR “periodontitis” OR “masticatory performance” OR “masticatory function” OR “chew*” OR “swallow” OR “halitos*” OR “xerostomia” OR “pulpitis”) ) | 191,735 |
|  | *Indexes=SCI-EXPANDED, SSCI, A&HCI, ESCI Timespan=All years* |  |
| #1 | TS=((“food” OR “drink*” OR “beverage*” OR “alcohol” OR “nutritional” OR “dietary”) NEAR/3 (“label*” OR “legislat*” OR “environment*” OR “access*” OR “availab*” OR “adverti*” OR “market*” OR “promot*” OR “policy” OR “price*” OR “afford*” OR “sponsor*” OR “cost*” OR “supply” OR “tax*” OR “retail” OR “outlet” OR “expos*” OR “packag*” OR “security” OR “insecurity” OR “regulat*” OR “divers*” OR “rules” OR “spatial access” OR “Product placement”) ) OR TS=(“foodscap*” OR “food desert”) | 202,274 |
|  | *Indexes=SCI-EXPANDED, SSCI, A&HCI, ESCI Timespan=All years* |  |

**EBSCO/CINAHL (29 April 2021)**

| **Search** | **CINAHL Query – April 29, 2021** | **Results** |
| --- | --- | --- |
| S6 | S5 NOT TI (“review”) | 320 |
| S6 | S1 AND (S2 OR S3 OR S4) | 340 |
| S4 | TI ((“oral” OR “mouth” OR “tooth” OR “dental” OR “gingiv*” OR “gum” OR “periodont*”) NEAR/3 (“microbio*” OR “flora” OR “microflora” OR “bacteri*” OR “microb*” OR “microorganism*” OR “micro-organism*”)) OR AB ((“oral” OR “mouth” OR “tooth” OR “dental” OR “gingiv*” OR “gum” OR “periodont*”) NEAR/3 (“microbio*” OR “flora” OR “microflora” OR “bacteri*” OR “microb*” OR “microorganism*” OR “micro-organism*”)) OR KW ((“oral” OR “mouth” OR “tooth” OR “dental” OR “gingiv*” OR “gum” OR “periodont*”) NEAR/3 (“microbio*” OR “flora” OR “microflora” OR “bacteri*” OR “microb*” OR “microorganism*” OR “micro-organism*”)) | 0 |
| S3 | (MH "Mouth Neoplasms+") OR (((MH "Neoplasms+") OR TI (“adenoma*” OR “anticarcinogen*” OR “blastoma*” OR “cancer*” OR “carcinogen*” OR “carcinom*” OR “carcinosarcoma*” OR “chordoma*” OR “malignan*” OR “melanom*” OR “mesenchymoma*” OR “metasta*” OR “neoplas*” OR “neuroma*” OR “nsclc” OR “oncogen*” OR “oncolog*” OR “paraneoplastic” OR “plasmacytoma*” OR “precancerous” OR “sarcoma*” OR “teratocarcinoma*” OR “teratoma*” OR “tumor*” OR “tumour*”) AND TI(“face” OR “facial*” OR “gingiva*” OR “head” OR “hypopharyn*” OR “jaw” OR “jaws” OR “laryn*” OR “lip” OR “lips” OR “mandib*” OR “mouth” OR “nasopharyn*” OR “neck” OR “nose” OR “oral*” OR “oropharyn*” OR “otorhinolaryn*” OR “palatal*” OR “palate” OR “palatum” OR “paranasal*” OR “paroti*” OR “pharyn*” OR “salivar*” OR “sublingual*” OR “submandib*” OR “throat” OR “tongue” OR “tonsil*”)) OR (((MH "Neoplasms+") OR AB (“adenoma*” OR “anticarcinogen*” OR “blastoma*” OR “cancer*” OR “carcinogen*” OR “carcinom*” OR “carcinosarcoma*” OR “chordoma*” OR “malignan*” OR “melanom*” OR “mesenchymoma*” OR “metasta*” OR “neoplas*” OR “neuroma*” OR “nsclc” OR “oncogen*” OR “oncolog*” OR “paraneoplastic” OR “plasmacytoma*” OR “precancerous” OR “sarcoma*” OR “teratocarcinoma*” OR “teratoma*” OR “tumor*” OR “tumour*”) AND AB (“face” OR “facial*” OR “gingiva*” OR “head” OR “hypopharyn*” OR “jaw” OR “jaws” OR “laryn*” OR “lip” OR “lips” OR “mandib*” OR “mouth” OR “nasopharyn*” OR “neck” OR “nose” OR “oral*” OR “oropharyn*” OR “otorhinolaryn*” OR “palatal*” OR “palate” OR “palatum” OR “paranasal*” OR “paroti*” OR “pharyn*” OR “salivar*” OR “sublingual*” OR “submandib*” OR “throat” OR “tongue” OR “tonsil*”)) OR (((MH "Neoplasms+") OR KW (“adenoma*” OR “anticarcinogen*” OR “blastoma*” OR “cancer*” OR “carcinogen*” OR “carcinom*” OR “carcinosarcoma*” OR “chordoma*” OR “malignan*” OR “melanom*” OR “mesenchymoma*” OR “metasta*” OR “neoplas*” OR “neuroma*” OR “nsclc” OR “oncogen*” OR “oncolog*” OR “paraneoplastic” OR “plasmacytoma*” OR “precancerous” OR “sarcoma*” OR “teratocarcinoma*” OR “teratoma*” OR “tumor*” OR “tumour*”) AND KW (“face” OR “facial*” OR “gingiva*” OR “head” OR “hypopharyn*” OR “jaw” OR “jaws” OR “laryn*” OR “lip” OR “lips” OR “mandib*” OR “mouth” OR “nasopharyn*” OR “neck” OR “nose” OR “oral*” OR “oropharyn*” OR “otorhinolaryn*” OR “palatal*” OR “palate” OR “palatum” OR “paranasal*” OR “paroti*” OR “pharyn*” OR “salivar*” OR “sublingual*” OR “submandib*” OR “throat” OR “tongue” OR “tonsil*”)) | 593,491 |
| S2 | (MH "Oral Health") OR (MH "Mouth Diseases+") OR TI (((“oral” OR “mouth” OR “tooth” OR “dental” OR “gingiv*” OR “gum” OR “periodont*”) NEAR/3 (“health” OR “disease*” OR “ulcer” OR “function” OR “hygiene” OR “quality of life” OR “status” OR “bleeding” OR “hemorr*” OR “haemorr*”)) OR ((“caries” OR “cariogenesis” OR “carious” OR “cavity” OR “plaque” OR “debris” OR “decay” OR “erosion” OR “Dmft” OR “Dmfs” OR “loss” OR “fluorosis”) NEAR/3 (“fissure” OR “dental” OR “dentin*” OR “tooth” OR “teeth” OR “enamel” OR “molar*” OR “root”)) OR (“malocclusion” OR “white spot lesion” OR “edentation” OR “periodontitis” OR “masticatory performance” OR “masticatory function” OR “chew*” OR “swallow” OR “halitos*” OR “xerostomia” OR “pulpitis” OR “noma” OR “oral thrush” OR “early childhood caries” OR “baby bottle syndrome” OR “nursing caries”)) OR AB (((“oral” OR “mouth” OR “tooth” OR “dental” OR “gingiv*” OR “gum” OR “periodont*”) NEAR/3 (“health” OR “disease*” OR “ulcer” OR “function” OR “hygiene” OR “quality of life” OR “status” OR “bleeding” OR “hemorr*” OR “haemorr*”)) OR ((“caries” OR “cariogenesis” OR “carious” OR “cavity” OR “plaque” OR “debris” OR “decay” OR “erosion” OR “Dmft” OR “Dmfs” OR “loss” OR “fluorosis”) NEAR/3 (“fissure” OR “dental” OR “dentin*” OR “tooth” OR “teeth” OR “enamel” OR “molar*” OR “root”)) OR (“malocclusion” OR “white spot lesion” OR “edentation” OR “periodontitis” OR “masticatory performance” OR “masticatory function” OR “chew*” OR “swallow” OR “halitos*” OR “xerostomia” OR “pulpitis” OR “noma” OR “oral thrush” OR “early childhood caries” OR “baby bottle syndrome” OR “nursing caries”)) OR KW (((“oral” OR “mouth” OR “tooth” OR “dental” OR “gingiv*” OR “gum” OR “periodont*”) NEAR/3 (“health” OR “disease*” OR “ulcer” OR “function” OR “hygiene” OR “quality of life” OR “status” OR “bleeding” OR “hemorr*” OR “haemorr*”)) OR ((“caries” OR “cariogenesis” OR “carious” OR “cavity” OR “plaque” OR “debris” OR “decay” OR “erosion” OR “Dmft” OR “Dmfs” OR “loss” OR “fluorosis) NEAR/3 (“fissure” OR “dental” OR “dentin*” OR “tooth” OR “teeth” OR “enamel” OR “molar*” OR “root”)) OR (“malocclusion” OR “white spot lesion” OR “edentation” OR “periodontitis” OR “masticatory performance” OR “masticatory function” OR “chew*” OR “swallow” OR “halitos*” OR “xerostomia” OR “pulpitis” OR “noma” OR “oral thrush” OR “early childhood caries” OR “baby bottle syndrome” OR “nursing caries”)) | 292,144 |
| S1 | (MH "Food Labeling") OR (MH "Food Packaging+") OR (MH "Food Supply") OR TI (((“food*” OR “drink*” OR “beverage*” OR “alcohol” OR “nutrition*” OR “diet*”) NEAR/3 (“label*” OR “legislat*” OR “environment*” OR “access*” OR “availab*” OR “adverti*” OR “market*” OR “promot*” OR “policy” OR “price*” OR “afford*” OR “sponsor*” OR “cost*” OR “supply” OR “tax*” OR “retail” OR “outlet” OR “packag*” OR “security” OR “insecurity” OR “regulat*” OR “divers*” OR “rules” OR “spatial access” OR “Product placement”)) OR (“foodscap*” OR “food desert”) OR AB (((“food*” OR “drink*” OR “beverage*” OR “alcohol” OR “nutrition*” OR “diet*”) NEAR/3 (“label*” OR “legislat*” OR “environment*” OR “access*” OR “availab*” OR “adverti*” OR “market*” OR “promot*” OR “policy” OR “price*” OR “afford*” OR “sponsor*” OR “cost*” OR “supply” OR “tax*” OR “retail” OR “outlet” OR “packag*” OR “security” OR “insecurity” OR “regulat*” OR “divers*” OR “rules” OR “spatial access” OR “Product placement”)) OR (“foodscap*” OR “food desert”) OR KW (((“food*” OR “drink*” OR “beverage*” OR “alcohol” OR “nutrition*” OR “diet*”) NEAR/3 (“label*” OR “legislat*” OR “environment*” OR “access*” OR “availab*” OR “adverti*” OR “market*” OR “promot*” OR “policy” OR “price*” OR “afford*” OR “sponsor*” OR “cost*” OR “supply” OR “tax*” OR “retail” OR “outlet” OR “packag*” OR “security” OR “insecurity” OR “regulat*” OR “divers*” OR “rules” OR “spatial access” OR “Product placement”)) OR (“foodscap*” OR “food desert”) | 9,847 |

# Appendix 1

# List with additional references used to refer to studies included in the systematic literature review

a Alattas M, Ross CS, Henehan ER, Naimi TS. Alcohol policies and alcohol-attributable cancer mortality in U.S. States. *Chem Biol Interact* 2020;315:108885.

b Choi SE, Wright DR, Bleich SN. Impact of Restricting Sugar-Sweetened Beverages From the Supplemental Nutrition Assistance Program on Children’s Health. *Am J Prev Med* 2021;60:276–84.

c Yang W, Carmichael SL, Shaw GM. Folic acid fortification and prevalences of neural tube defects, orofacial clefts, and gastroschisis in California, 1989 to 2010. *Birth Defects Res Part A - Clin Mol Teratol* 2016;106:1032–41.

d Yazdy MM, Honein MA, Xing J. Reduction in orofacial clefts following folic acid fortification of the U.S. grain supply. *Birth Defects Res Part A - Clin Mol Teratol* 2007;79:16–23.

e Maupomé G, Karanja N, Ritenbaugh C, Lutz T, Aickin M, Becker T. Dental caries in American Indian toddlers after a community-based beverage intervention. *Ethn Dis* 2010;20:444–50.

f Tellez M, Sohn W, Burt BA, Ismail AI. Assessment of the Relationship between Neighborhood Characteristics and Dental Caries Severity among Low-Income African-Americans: A Multilevel Approach. *J Public Health Dent* 2006;66:30–36.

g Urwannachotima N, Hanvoravongchai P, Ansah JP. Sugar-sweetened Beverage Tax and Potential Impact on Dental Caries in Thai Adults: An Evaluation Using the Group Model Building Approach. *Syst Res Behav Sci* 2019;36:87–99.

h Urwannachotima N, Hanvoravongchai P, Ansah JP, Prasertsom P, Koh VRY. Impact of sugar-sweetened beverage tax on dental caries: A simulation analysis. *BMC Oral Health* 2020;20:1–12.

i Kaewkamnerdpong I, Krisdapong S. Oral diseases associated with condition-specific oral health-related quality of life and school performance of Thai primary school children: A hierarchical approach. *Community Dent Oral Epidemiol* 2018;46:270–79.

j Sowa PM, Keller E, Stormon N, Lalloo R, Ford PJ. The impact of a sugar-sweetened beverages tax on oral health and costs of dental care in Australia. *Eur J Public Health* 2019;29:173–77.

k Jiang H, Livingston M, Room R, Gan Y, English D, Chenhall R. Can public health policies on alcohol and tobacco reduce a cancer epidemic? Australia’s experience. *BMC Med* 2019;17:1–14.

l Schwendicke F, Thomson WM, Broadbent JM, Stolpe M. Effects of taxing sugar-sweetened beverages on caries and treatment costs. *J Dent Res* 2016;95:1327–32.

m Jevdjevic M, Wijn SRW, Trescher AL, Nair R, Rovers M, Listl S. Front-of-Package Food Labeling to Reduce Caries: Economic Evaluation. *J Dent Res* 2021;100:472–78.

n Ghimire N, Rao A. Comparative evaluation of the influence of television advertisements on children and caries prevalence. *Glob Health Action* 2013;6. doi:10.3402/gha.v6i0.20066.

o Somasundaram R, Rangeeth BN, Moses J, Sivakumar S. Comparison of the source of introduction to cariogenic food substance and caries prevalence in children. *J Clin Diagnostic Res* 2014;8:138–40.

p Freeman R, Oliver M, Bunting G, Kirk J, Saunderson W. Addressing children’s oral health inequalities in Northern Ireland: A research-practice-community partnership initiative. *Public Health Rep* 2001;116:617–25.

q Freeman R, Oliver M. Do school break-time policies influence child dental health and snacking behaviours? An evaluation of a primary school programme. *Br Dent J* 2009;206:619–25.

r Edasseri A, Barnett TA, Kâ K, Henderson M, Nicolau B. Oral Health–Promoting School Environments and Dental Caries in Québec Children. *Am J Prev Med* 2017;53:697–704.

s Jamel H, Plasschaert A, Sheiham A. Dental caries experience and availability of sugars in Iraqi children before and after the United Nations sanctions. *Int Dent J* 2004;54:21–25.

t Hernández-F M, Cantoral A, Colchero MA. Taxes to Unhealthy Food and Beverages and Oral Health in Mexico: An Observational Study. *Caries Res* 2021;55:183–92.

u Jevdjevic M, Trescher AL, Rovers M, Listl S. The caries-related cost and effects of a tax on sugar-sweetened beverages. *Public Health* 2019;169:125–32.

v Thornley S, Marshall R, Reynolds G, Koopu P, Sundborn G, Schofield G. Low sugar nutrition policies and dental caries: A study of primary schools in South Auckland. *J Paediatr Child Health* 2017;53:494–99.

w Briggs ADM, Mytton OT, Kehlbacher A, et al. Health impact assessment of the UK soft drinks industry levy: a comparative risk assessment modelling study. *Lancet Public Heal* 2017;2:e15–22.
